# Mr. Henry Gray

**Published:** 1861-09

**Authors:** 


MR. HENRY GRAY, so well known in this country by his excellent work on anatomy, died in London on the 12th of June, at the early age of thirty four. At the time of his death he was lecturer on anatomy at, and surgeon to, St. George's Hospital. The loss of so valuable a man must be deeply deplored by the profession everywhere.

